# Mechanism of Exosomes Involved in Osteoimmunity Promoting Osseointegration Around Titanium Implants With Small-Scale Topography

**DOI:** 10.3389/fbioe.2021.682384

**Published:** 2021-07-15

**Authors:** Ting Zhang, Mengyang Jiang, Xiaojie Yin, Peng Yao, Huiqiang Sun

**Affiliations:** ^1^Department of Prosthodontics, School and Hospital of Stomatology, Cheeloo College of Medicine, Shandong University & Shandong Key Laboratory of Oral Tissue Regeneration & Shandong Engineering Laboratory for Dental Materials and Oral Tissue Regeneration, Jinan, China; ^2^School of Mechanical Engineering, Shandong University, Jinan, China

**Keywords:** exosomes, microRNA, osteoimmunity, titanium, osseointegration

## Abstract

Exosomes are nanoscale extracellular vesicles. Several studies have shown that exosomes participate in intercellular communication and play a key role in osseointegration. However, it is unclear whether exosomes and their contents participate in the communication between the immune and skeletal systems in the process of osseointegration. In this study, we obtained smooth titanium disks by polishing and small-scale topography titanium disks by sandblasted large-grit acid-etched (SLA) technology combined with alkali thermal reaction. After stimulating mouse RAW264.7 cells with these two kinds of titanium disks, we co-cultured the MC3T3-E1 cells and the RAW264.7 cells, obtained and identified the exosomes derived from RAW264.7 cells, and studied the effect of the osteoimmune microenvironment and the exosomes on the osseointegration of mouse MC3T3-E1 cells. Cell counting kit-8 (CCK-8), real time quantitative PCR, western blotting, alizarin red staining, and quantitative and confocal fluorescence microscopy were used to study the effects of exosomes on MC3T3-E1 cells; RNA sequencing and correlation analysis were performed. We found that the osteoimmune microenvironment could promote the osseointegration of MC3T3-E1 cells. We successfully isolated exosomes and found that RAW264.7 cell-derived exosomes can promote osteogenic differentiation and mineralization of MC3T3-E1 cells. Through RNA sequencing and gene analysis, we found differentially expressed microRNAs that targeted the signal pathways that may be related, such as mTOR, AMPK, Wnt, etc., and thus provide a reference for the mechanism of osteoimmunue regulation of implant osseointegration. The study further elucidated the mechanism of implant osseointegration and provided new insights into the effect of exosomes on implant osseointegration, and provided reference for clinical improvement of implant osseointegration and implant success rate.

## Introduction

In recent years, implant dentures have gradually become an important treatment option for missing teeth. Titanium and titanium alloys have good biocompatibility and mechanical properties and are among the most widely used implant materials in clinics (Xue et al., 2020). However, because titanium is an inert metal with no biological activity, it can easily cause a host inflammatory reaction that can even progress to chronic inflammation, delaying implant osseointegration (Dar et al., 2018). Therefore, many techniques have been applied for titanium surface modification (Dohan Ehrenfest et al., 2010), such as sandblasted large-grit acid etching (SLA) and anodization (Loi et al., 2016; Miron and Bosshardt, 2016). In our previous study, we combined SLA technology with an alkali thermal reaction to construct titanium implants with small-scale topography. It was found that, small-scale topography promoted MC3T3-E1 cell proliferation and osteogenic differentiation better than the polished smooth surface, the micro-surface obtained by SLA technology and the nano-surface obtained by alkali thermal reaction (Yang et al., 2020).

The process of osseointegration of implants involves the coordinated operation of the immune and skeletal systems, namely osteoimmunity (Dar et al., 2018). Macrophages can be differentiated into resident cells or myeloid precursor cells (mainly monocytes) and reside in the bone. The interaction between macrophages and osteocytes is crucial for bone formation and repair (Pieters et al., 2019). Some studies have found that different implant surface morphologies can induce macrophages to polarize to the pro-inflammatory M1 phenotype or anti-inflammatory M2 phenotype (Luu et al., 2015; Quan et al., 2020). In our previous study, we found that the small-scale topography can stimulate RAW264.7 cells to polarize to anti-inflammatory M2 phenotype and regulate the osteoimmune microenvironment to an anti-inflammatory environment, which is more conducive to implant osseointegration (Yang et al., 2020).

Exosomes are nano-sized vesicles that are secreted by most cells. They were first found in reticulocytes in 1983 and named exosome in 1987 (Pan and Johnstone, 1983). The diameter of exosomes is in the range of 30–150 nm, with a lipid bilayer structure (Mathieu et al., 2019); exosomes can be directly absorbed by target cells and affect the phenotype of receptor cells (Rani et al., 2015; Yang et al., 2017). Therefore, they play an important role in cell communication and have attracted increasing attention. Wei et al. (2019) found that the expression of alkaline phosphatase (ALP) and BMP-2 markers of early osteoblast differentiation was significantly increased by using BMP-2/macrophage- derived exosomes to modify titanium nanotube implants, which confirmed that the combination of titanium nanotubes and BMP-2/macrophage-derived exosomes could promote bone formation. Xiong et al. (2020) found that M2 macrophage-derived exosome microRNA-5106 could induce the osteogenic differentiation of bone marrow mesenchymal stem cells.

Exosomes play an important role in target cells, mainly through intercellular communication and the delivery of key bioactive factors. However, it is not clear whether exosomes participate in osteoimmunity and influence osteointegration around titanium implants with small-scale topography. Furthermore, the gene information and function of macrophage-derived exosomes have not been fully clarified. Therefore, we studied the effect of macrophage-derived exosomes stimulated by small-scale topography of titanium implants on MC3T3-E1 cells and screened key microRNAs in exosomes, to further explore the mechanism of macrophages stimulated by exosomal contents in small-scale topography titanium disks on MC3T3-E1 cells, and to provide reference for exploring the effect of osteoimmunity on osseointegration.

## Materials and Methods

### Preparation and Characterization of Titanium Disk

Ti6Al4V disks with a diameter of 19.5 mm and thickness of 1 mm (Taizhou Yutai Metal Materials Co., Ltd., Jiangsu, China) were used and polished to an average roughness of 0.2 mm and a thickness of 0.01 mm. The polishing process of the disks was listed in Supplementary File 1. The 60 mesh alumina particles (Gongyi Baolai Water Treatment Material Factory, Henan, China) were sprayed on the polished titanium plate surface at a spray angle of 90° with a spray distance of no more than 5 cm. When the surface of the titanium disk was uniformly gray, it was removed and immersed in 0.5% hydrofluoric acid solution for 15 min at 25°C. For alkali thermal treatment, the titanium disk was immersed in a 10 mol/L sodium hydroxide solution and treated at 80°C for 24 h. All the titanium disks were placed into a 5% concentrated cleaning solution (micro-90, International Products Company, New York, United States), anhydrous ethanol, distilled water, ultrasonic vibration cleaning for 5 min, and air dried at room temperature for standby.

For the surface characterization of the titanium disk, the surface morphology was observed using a cold field emission scanning electron microscope (Carl Zeiss, Germany) and 3D laser scanning microscope (VK-X200 K, Japan). The surface contact angle was measured by the suspension drop method with 2 μL artificial saliva, and the average surface roughness (RA) of the titanium disk was measured using a Wyko nt9300 optical profiler (Veeco, United States). The surface composition of titanium disks was analyzed by X-ray photoelectron spectrometer (Thermo Fisher Scientific, United States).

### Cell Culture

RAW264.7 cells, a widely used mouse derived macrophage cell line, were provided by Shandong Key Laboratory of oral tissue. Mouse embryonic osteoblast precursor cells (MC3T3-E1 cells) were purchased from the Cell Bank of the Chinese Academy of Sciences (Shanghai, China). The complete medium was α-minimum essential medium (α-MEM, Hyclone, United States), in which 10% fetal bovine serum (FBS, Hyclone, United States) was added. The culture medium contained double antibodies (100 IU/mL penicillin G and 100 μg/mL streptomycin, Solebo, China). The cells were cultured at 37°C in a 5% CO_2_ incubator, and the medium was changed on alternate days. Osteogenesis induction medium (OM) consisted of complete medium supplemented with 50 mg/L ascorbic acid (Sigma Aldrich), 10 mmol/L β-glycerophosphate (Sigma Aldrich), and 10 nmol/L dexamethasone (Sigma Aldrich). The supernatant of RAW264.7 cell culture medium was centrifuged at 1000 × *g* for 5 min, and then the conditioned medium (CM) was prepared by adding 20% FBS osteogenic induction medium.

### Exosome Isolation

The supernatant of RAW264.7 cell culture medium was centrifuged at 1000 × *g* for 5 min, and then 20 mL of each group was added into a 50 mL centrifuge tube. PBS was added to bring it to a total of 40 mL in each tube, centrifuged at 4°C for 10 min at 300 × *g*, and the supernatant was transferred to a new 50 mL, the supernatant was transferred to a centrifuge tube (40 mL), matched with the ultracentrifuge, and centrifuged at 4°C for 30 min at 10,000 × *g*. The supernatant was transferred to a centrifuge tube (40 mL), matched with the ultracentrifuge, and centrifuged at 4°C for 70 min at 10,0000 × *g*. The exosomes were identified by transmission electron microscopy, nanoparticle tracking analysis, and western blotting.

### Nanoparticle Tracking Analysis (NTA)

The sample pool was cleaned with deionized water, the instrument was calibrated with polystyrene microspheres (110 nm), and the sample pool was cleaned with 1 × PBS buffer; the exosome solution was diluted with 1 × PBS buffer and injected for detection. Each sample was detected three times, and the data were processed and mapped using the Origin software.

### CCK-8 Detection

Cell proliferation was examined using the cell counting kit 8 (CCK-8; Dojindo, Tokyo, Japan). MC3T3-E1 cells were treated with osteogenic induction medium or CM. On days 1, 3, 5, and 7, the original medium was replaced with CCK-8 reagent and complete medium at a ratio of 1:10. MC3T3-E1 cells were incubated at 37°C for 1 h, and the absorbance value was measured at 450 nm.

### Alizarin Red S Staining and Cetylpyridinium Chloride Determination

MC3T3-E1 cells were fixed with 4% paraformaldehyde for 30 min and then incubated with alizarin red S (Sigma Aldrich) for 10 min. After washing with deionized water, the calcium deposition was observed using an optical microscope. Cetylpyridinium chloride (10%; Sigma Aldrich) was used for quantification, and the absorbance value was determined at 562 nm.

### Real Time Fluorescent Quantitative PCR (qRT-PCR)

Total RNA was extracted using TRIzol reagent (Invitrogen, NY, United States). cDNA was synthesized using the PrimeScript RT Master Mix Kit (Takara Biotechnology, China). GAPDH was selected as the internal reference gene, and the relative gene expression was calculated by the “2−△△CT” method. The primer sequences are listed in Table 1.

**TABLE 1:** List of primers used in this study for qRT-PCR.

Genes	Primers	Sequences (5′–3′)
GAPDH	Forward	AGGTCGGTGTGAACGGATTTG
	Reverse	TGTAGACCATGTAGTTGAGGTCA
IL-1β	Forward	GTGTCTTTCCCGTGGACCTT
	Reverse	AATGGGAACGTCACACACCA
IL-10	Forward	GCTCTTGCACTACCAAAGCC
	Reverse	CTGCTGATCCTCATGCCAGT
Runx2	Forward	GGGACTGTGGTTACCGTCAT
	Reverse	ATAACAGCGGAGGCATTTCG
Collagen I	Forward	CCCTGGTCCCTCTGGAAATG
	Reverse	GGACCTTTGCCCCCTTCTTT

### Western Blot

Cells were lysed with Ripa buffer (Wanleibio, China) containing 10 mM protease inhibitor (PMSF; Wanleibio, China). The lysate was centrifuged at 12,000 rpm and 4°C for 10 min. The supernatant was separated, and the protein concentration was determined using a BCA protein concentration assay kit (Wanleibio, China). Equivalent amounts of protein (40 g) were added to the 8%-15% SDS-PAGE gel and then transferred to a PVDF membrane (Millipore, Billerica, United States). The membrane was sealed in 5% skimmed milk powder solution for 1 h, and then incubated with the following primary antibodies: runt-related transcription factor 2 (Runx2, wl03358; Wanleibio, China), Collagen I (wl0088; Wanleibio, China), β-actin (wl01845; Wanleibio, China), CD9 (ab92726, Abcam, United Kingdom), CD63 (ab216130, Abcam, United Kingdom), and TSG101 (ab125011, Abcam, United Kingdom) at 4°C overnight. After washing with TBST four times, the membrane was incubated with sheep anti-rabbit IgG HRP (wla023; Wanleibio, China) at room temperature for 45 min. An ECL detection kit (Wanleibio, China) was used to visualize the protein bands. Proteins on the same membrane were compared quantitatively by determining the optical density of the target strip using a gel image processing system (Gel-Pro-Analyzer software, Media Cybernetics, United States).

### Exosome RNA Sequencing and Gene Analysis

According to the standard procedure provided by Illumina (San Diego, United States), the miRNA sequencing library was prepared using the TruSeq Small RNA Sample Prep Kit (Illumina, San Diego, United States). After library preparation, the constructed library was sequenced using Illumina HiSeq2000/2500, and the reading length was 1 × 50 bp. Clean reads were obtained from original data after quality control. The 3′-linker was removed from the clean reads, and the length of the 3′ linker was screened. Sequences with a base length of 18–25 nt (plant) or 18–26 nt (animal) were retained. The remaining sequences were then aligned to various RNA database sequences (excluding miRNA), such as mRNA database, Rfam database (including rRNA, tRNA, snRNA, snoRNA, etc.) and RepBase database (repetitive sequence database), and filtered. Finally, the data obtained were valid. MicroRNA data analysis software Acgt101 Mir Program (LC Sciences, Houston, Texas, United States) was used to analyze the differentially expressed miRNAs.

### Bioinformatics Analysis

The number of genes annotated for each function or pathway corresponding to the target genes which were corresponded to the differentially expressed miRNAs was counted, and then the hypergeometric test was applied to determine the number of genes in the Gene ontology (GO) and Kyoto Encyclopedia of Genes and Genomes (KEGG) database corresponding to the target gene mRNAs which were corresponded to all the selected microRNAs. Compared all the gene in the database above (all genes with functional annotation, or all miRNA target genes with functional annotation), the significantly enriched genes or pathways were selected under the standard that *p*-value less than or equal to 0.05. The formula for calculating the *p*-value is as follows. $P=1-\sum_{i=0}^{s-1}\frac{\left(\begin{array}{cc} B & T\,B-B\\ i & T\,S-i \end{array}\right)}{\left(\begin{array}{c} T\,B\\ T\,S \end{array}\right)}$ (S: the number of genes with significant differences matched to a single GO/KEGG; TS: the total number of genes with significant differences; B: number of genes matched to a single GO/KEGG; TB: total number of genes.) We used the tool available on this link to draw: https://www.omicstudio.cn/tool?order=complex.

### Statistical Analysis

Quantitative results are expressed as mean ± standard deviation (SD). The experiment was repeated independently at least three times. Univariate analysis of variance and multiple t-tests were used for statistical evaluation, and *p* < 0.05 was set as a statistically significant threshold.

## Results

### Surface Characterization of Titanium Disks

**FIGURE 1:**
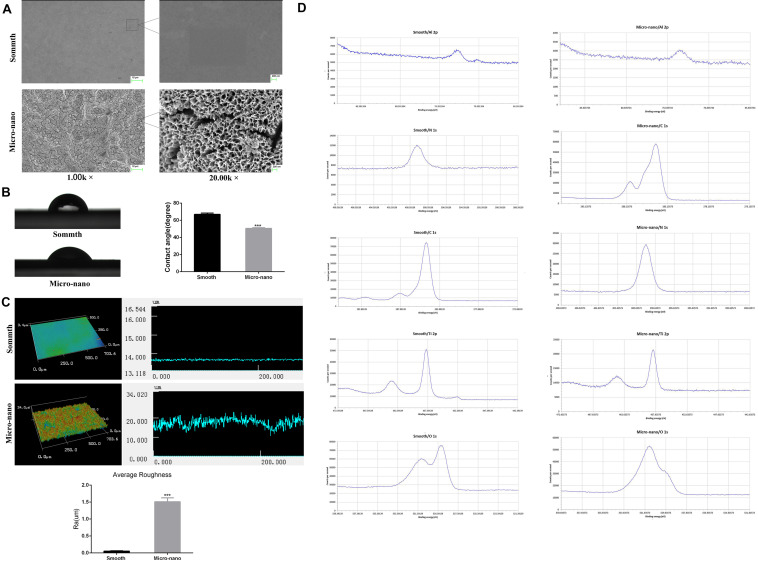
Surface characterization of materials. **(A)** The surface morphology of titanium disk was observed by using a scanning electron microscope. **(B)** Contact angle test. **(C)** Three-dimensional structure diagram observation and roughness test (Yang et al., 2020). **(D)** XPS analysis (smooth: smooth titanium disk; micro-nano: small-scale topography titanium disk. ***p < 0.001).

Figure 1A and Supplementary File 2 exhibits that the surface of smooth titanium disk is flat, without scratches; under the low power microscope, the surface of small-scale topography titanium disk is rough, no residual alumina particles are found, and a large number of pits can be seen. The pits are in the form of a multilevel continuous superposition. The secondary depression with a diameter of 2–8 μm is superimposed on the surface of the first depression with a diameter of 10–50 μm, and nanopores with a diameter of approximately 50–200 nm can be viewed under a high-power microscope.

Figure 1B shows the surface droplet morphology and average surface hydrophilicity of the two types of titanium disks. Using 2 μL artificial saliva as a wetting agent, the contact angle of the small-scale topography titanium disk was significantly lower than that of the smooth titanium disk (*p* < 0.05). The superficial 3D microstructure and roughness are shown in Figure 1C. The average surface roughness, Ra, of the two kinds of titanium disks was further observed and compared using an optical profiler. In comparison with the smooth titanium disk, the average surface roughness of small-scale topography titanium disk increased significantly (*p* < 0.05; Figure 1C, Supplementary File 3), which is consistent with the results of scanning electron microscope (Yang et al., 2020). The analysis results of the surface composition of the titanium disc are shown in the Figure 1D and Supplementary Files 4, 5. All the results above proved that the titanium disk obtained by SLA technology combined with alkali heat treatment was small-scale topography, which increased the surface roughness of the titanium disk and was more conducive to osseointegration.

### Small-Scale Topography Titanium Disk Induces Macrophages to Polarize to Anti-inflammatory M2 Type

**FIGURE 2:**
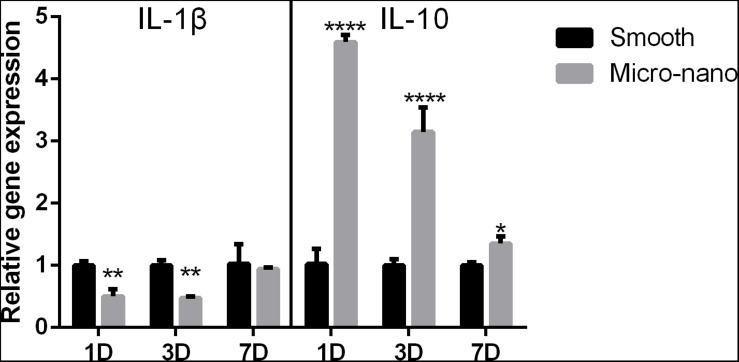
Gene expression of RAW264.7 cells on different titanium disks. (smooth, smooth titanium disk; micro-nano, small-scale topography titanium disk. *p < 0.05, **p < 0.01, ****p < 0.0001).

After RAW264.7 cells were cultured on the surface of smooth and small-scale topography titanium disks, the expression of inflammation-related genes was detected (Figure 2). Compared to that on the surface of the smooth titanium disks, the expression level of the anti-inflammatory related gene IL-10 on the surface of small-scale topography titanium disk was increased and the expression level of the pro-inflammatory related gene IL-1β was decreased, with statistical significance, which was consistent with our previous research (Yang et al., 2020), and proved that small-scale topography titanium disk could stimulate the differentiation of RAW264.7 cells into M2 type to form an anti-inflammatory immune microenvironment.

### M2 Type RAW264.7 Cells Can Induce Osteogenic Differentiation of MC3T3-E1 Cells, and Exosomes May Play a Key Role in It

**FIGURE 3:**
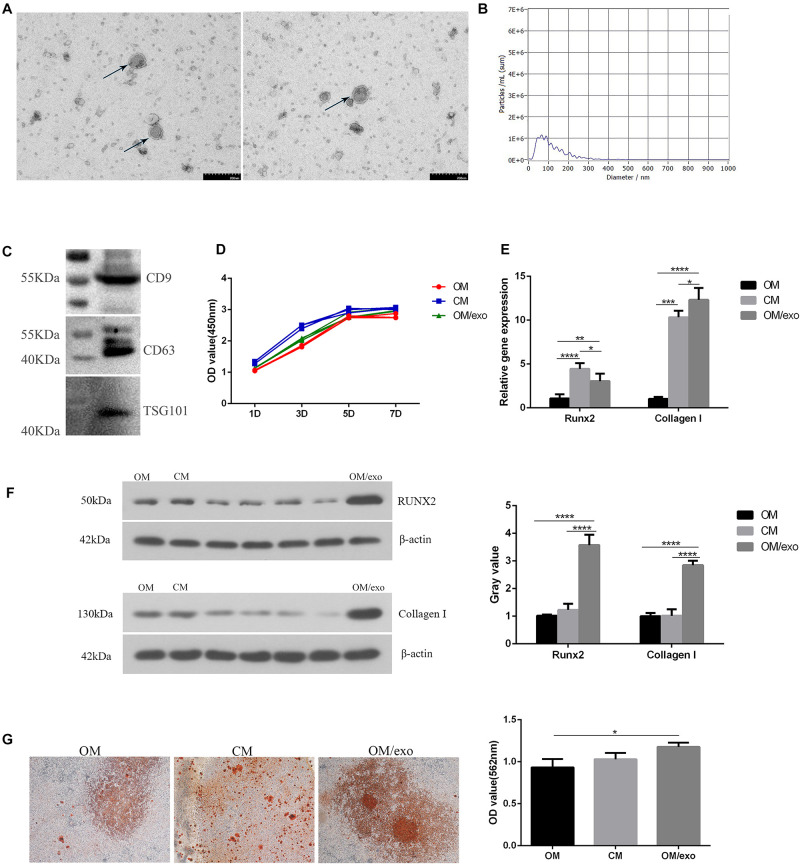
Identification of exosomes and their effects on osteogenic differentiation of MC3T3-E1 cells. **(A)** TEM showed the shape of exosomes; **(B)** NTA showed the diameter of exosomes; **(C)** Western blot showed the expression of exosomes surface marker protein CD9, CD63, and TSG101; **(D)** CCK-8 showed cell proliferation; **(E)** qRT-PCR showed the expression of osteogenic related genes Runx2, Collagen I in MC3T3-E1 cells; **(F)** Western blot showed the expression of osteogenic related genes Runx2 and Collagen I in MC3T3-E1; **(G)** The mineralization of MC3T3-E1 cells was detected by alizarin red. [OM, osteogenic induction medium; CM (osteogenic induction medium + RAW264.7 cell culture medium), OM/exo: CM with RAW264.7 cell-derived exosomes (*p < 0.05; **p < 0.01; ***p < 0.001; ****p < 0.0001)].

In order to study the effect of the osteoimmune microenvironment formed by M2 type RAW264.7 cells on MC3T3-E1 cells, we co-cultured the two types of cells. The CCK-8 result showed that compared with OM, CM could promote MC3T3-E1 cells proliferation better (Figure 3D). The qRT-PCR and Western blot results showed that compared with OM, CM enhanced the expression of Collagen I and Runx2 in MC3T3-E1 cells (Figures 3E,F). AND the alizarin red staining and semi-quantitative studies showed that the mineral deposition of cells of CM was increased (Figure 3G). We speculated that the exosomes played an important role in the process that osteoimmunity promoted the osteogenic differentiation of MC3T3-E1 cells. Therefore, the exosomes derived from RAW264.7 cells stimulated by the two kinds of titanium disks were separated and studied.

First, we characterized the exosomes derived from RAW264.7 cell. Transmission electron microscopy (TEM) results showed that the separated vesicles had a round bilayered membrane structure (Figure 3A). NTA results showed that the peak diameter of vesicles was 63.3 nm, accounting for 64.9% of the total area. The average diameter of vesicles was 117.1 nm, the distribution of vesicles was in the range of 30–200 nm (Figure 3B); the expression of exosome surface proteins CD9, CD63 and TSG101 was detected by western blot (Figure 3C); the above results demonstrated that the vesicles isolated from RAW264.7 cells were exosomes.

Next, we studied the effect of the exosomes on osteogenic differentiation of MC3T3-E1 cells. The CCK-8 assay was used to study the effect of cell proliferation (Figure 3D). In comparison with the control group, exosomes promoted the proliferation of MC3T3-E1 cells, but the effect was not as pronounced as that of the CM. qRT-PCR and western blotting were used to detect the expression of osteogenesis-related marker genes and proteins (Figures 3E,F). The results showed that compared to OM and CM, the expression of Collagen I and Runx2 in exosome-stimulated MC3T3-E1cells had significantly increased. Alizarin red staining and semi-quantitative studies showed that the effect of exosomes on promoting extracellular mineralization was similar to that of the MC3T3-E1 cells in CM (Figure 3G). The above studies had proved that exosomes played a key role in the process of oateoimmunity to promote osseointegration.

### Differentially Expressed MicroRNA Detected in RAW264.7 Cell-Derived Exosomes Stimulated by Smooth Titanium Disk and Small-Scale Topography Titanium Disk

**FIGURE 4:**
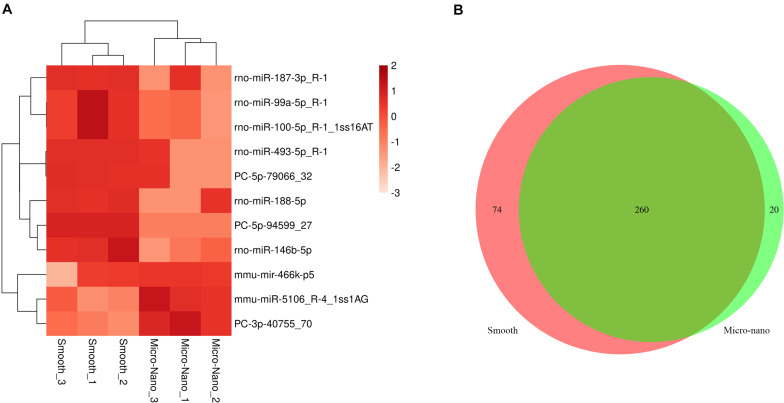
Differentially expressed microRNAs were detected in RAW264.7 cell-derived exosomes stimulated by smooth and small-scale topography titanium disks. **(A)** The heat map of differentially expressed exosome miRNAs; **(B)** Venn map showed that there were 260 common miRNAs and 20 specific miRNAs in the differentially expressed miRNAs.

**FIGURE 5:**
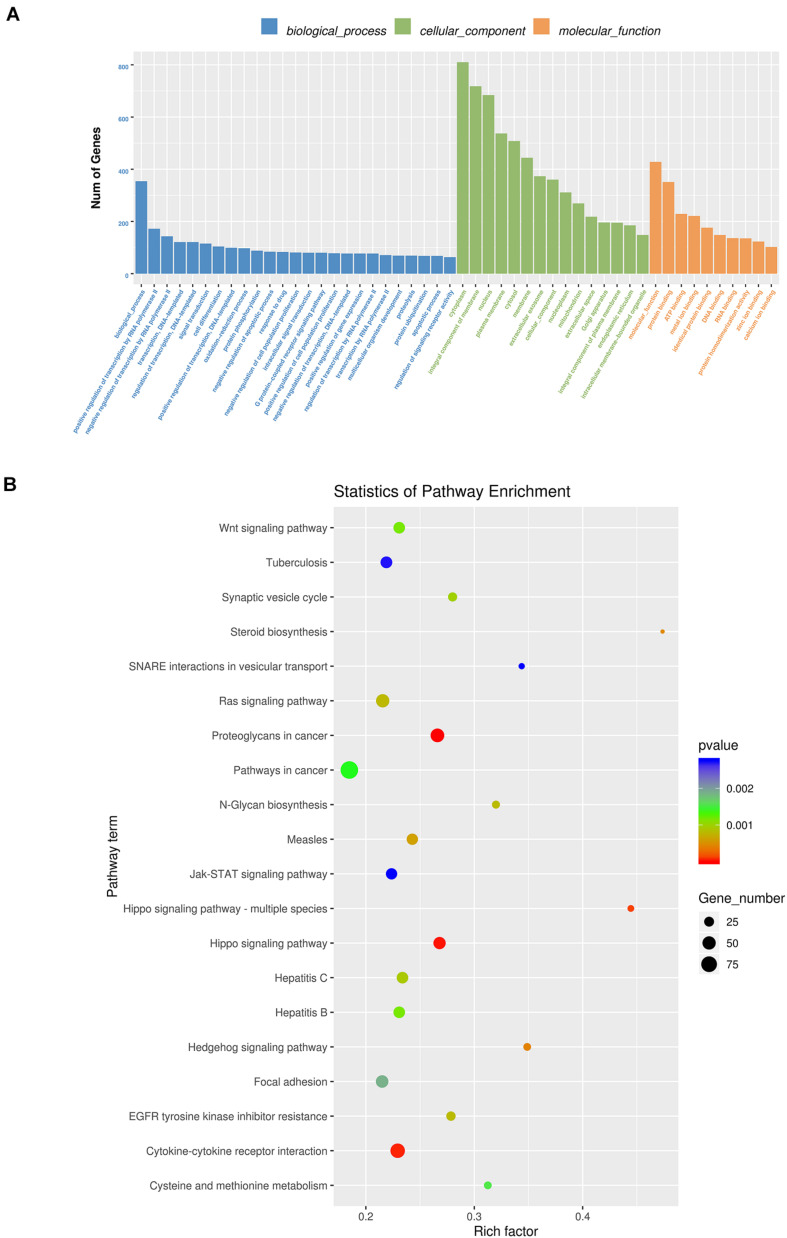
GO analysis and Kyoto Encyclopedia of Genes and Genomes (KEGG) pathway analysis. **(A)** GO analysis enrichment map of biological process, cell composition and molecular function; **(B)** KEGG pathway analysis enrichment map (Blue means 0.002 < *p* < 0.05; Green means 0.002 < *p* < 0.001; Red means *p* < 0.001).

**FIGURE 6:**
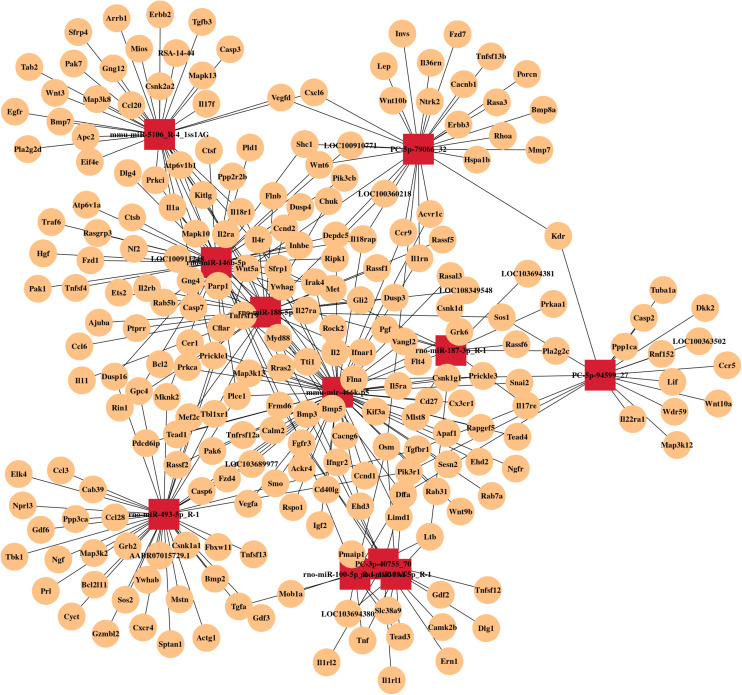
MicroRNA mRNA network. The relationship between differentially expressed miRNAs and their target genes.

By RNA sequencing, we analyzed the differentially expressed microRNAs in RAW264.7 cell-derived exosomes stimulated by two kinds of titanium disks. The abscissa of the heat map represents the sample cluster, and the ordinate represents the gene cluster (Figure 4A). A total of 260 mature miRNAs and 20 specific miRNAs were expressed in RAW264.7 cell-derived exosomes (Figure 4B). Exosome microRNAs can regulate related signaling pathways by targeting downstream target genes, thereby affecting successful differentiation. According to further GO and KEGG pathway analyses, differentially expressed miRNAs are involved in most biological processes, and are mainly regulated in the Hippo signaling pathway, Wnt signaling pathway, and mTOR signaling pathway (Figures 5A,B). Table 2 summarizes and lists the 11 upregulated and downregulated miRNAs and generates the regulatory network of these 11 miRNAs (Figure 6). Some genes involved in cell proliferation and differentiation are involved, which helps to further clarify the mechanism of differential expression of microRNAs in regulating osteoimmunity and promoting osseointegration.

**TABLE 2:** Differentially expressed microRNAs.

miR name	miR seq	Log2 ratio	Up/down	*P*-value
mmu-miR-5106_R-4_1ss1AG	GGGTCTGTAGCTCAGTTGG	0.60	Up	1.22E-02
mmu-mir-466k-p5	GTGTGTGTGTGTGTGTGTG	2.02	Up	1.36E-02
PC-3p-40755_70	GCGTGAGGAAGGAGGGGA	1.39	Up	2.43E-02
PC-5p-94599_27	GCATTGCCGGGTAGCTAA	-inf	Down	8.77E-03
rno-miR-493-5p_R-1	TTGTACATGGTAGGCTTTCAT	−2.72	Down	1.65E-02
rno-miR-146b-5p	TGAGAACTGAATTCCATAGGCTGT	−1.31	Down	2.14E-02
rno-miR-99a-5p_R-1	AACCCGTAGATCCGATCTTGT	−0.27	Down	3.27E-02
rno-miR-100-5p_R-1_1ss16AT	AACCCGTAGATCCGATCTTGT	−0.27	Down	3.27E-02
PC-5p-79066_32	GAGGAGTACTAGTCGGCA	−2.35	Down	3.61E-02
rno-miR-188-5p	CATCCCTTGCATGGTGGAGGG	−2.72	Down	4.16E-02
rno-miR-187-3p_R-1	TCGTGTCTTGTGTTGCAGCCG	−2.24	Down	4.79E-02

## Discussion

In this study, we used titanium disks to stimulate RAW264.7 cells to polarize into anti-inflammatory M2 phenotype, and isolated exosomes from RAW264.7 cells. We found that exosomes induced osteogenic differentiation, and mineralization of MC3T3-E1 cells. Meanwhile, we summarized the expression of microRNA of RAW264.7 cell-derived exosomes, which could provide a basis for exploring the mechanism of RAW264.7 cell-derived exosomes involved in the osteogenic differentiation of MC3T3-E1 cells.

Titanium and titanium alloys are the most widely used dental implant materials in clinics. The surface morphology of the implant has an important influence on osseointegration. A large number of studies have shown that the small-scale topography of the implant surface is more conducive to the biological behavior of osteoblasts than the simple micro-or nano- morphology (Wang et al., 2012, 2016; Ren et al., 2018). Micro-morphology can improve the mechanical properties of the implant by increasing the surface area of the implant and enhancing the mechanical chimerism between the implant and the bone cells (Dohan Ehrenfest et al., 2010). Nano-morphology can regulate the behavior of the implant by regulating the information transmission between cells (Chen et al., 2018). Compared to the traditional SLA technology, small-scale topography has a stronger ability to form hydroxyapatite *in vitro*, which can better promote the adhesion and extension of bone cells and promote osteogenesis cell proliferation and differentiation (Wang et al., 2012, 2016; Ren et al., 2018; Yang et al., 2020).

During osseointegration, there is a close relationship between the immune system and skeletal system. The immune system plays a key role in tissue repair and regeneration (Forbes and Rosenthal, 2014); macrophages and myeloid heterogeneous immune cells play an important role in the process of osseointegration. They can polarize to the pro-inflammatory M1 phenotype or anti-inflammatory M2 phenotype according to different stimuli (Gordon and Taylor, 2005; Gordon and Martinez, 2010; Michalski and McCauley, 2017). Many studies have shown that immune cells can be affected by various factors of the implant, including surface morphology (Fink et al., 2008; Spiller et al., 2009). In our previous study, we found that the small-scale topography titanium disk can stimulate RAW264.7 cells to polarize to the M2 phenotype, secrete IL-10 and VEGF, regulate the immune environment, and promote the osteogenic differentiation of MC3T3-E1 cells (Yang et al., 2020).

Exosome biogenesis is a dynamic but highly ordered process involving two invasions of the plasma membrane and the formation of intraluminal vesicles (ILVs) and intracellular multivesicular bodies (MVBs) (Li et al., 2019; Kalluri and LeBleu, 2020). Then, MVBs fuse with lysosomes to degrade, or with the plasma membrane to release ILV into the extracellular environment and become exosomes (van Niel et al., 2018; Mathieu et al., 2019), which then actively participate in the functional changes of many cells. The nature and content of exosomes are cell type-specific (Kalra et al., 2016), which are usually influenced by the physiological or pathological state of donor cells, the stimulation, and the molecular mechanism of biogenesis (Minciacchi et al., 2015). Studies have shown that many cell types and molecular mechanisms contribute to the coupling between bone resorption and bone formation (Sims and Martin, 2014). Exosomes from mononuclear phagocytes are most likely to play a role in maintaining bone homeostasis (Silva et al., 2017). Belonging to Runx family, expressed in osteoblasts, Runx2 is responsible for activating osteoblast differentiation marker genes, and plays a key role in the process of osteogenic differentiation (Vimalraj et al., 2015). Ekström et al. (2013) showed that LPS-stimulated monocytes can communicate with mesenchymal stem cells through exosomes, which can increase the expression of Runx2 and BMP-2 in mesenchymal stem cells. This is considered to be an intercellular signal transduction mode from the stages of injury and inflammation till bone regeneration. Qin et al. (2016) confirmed that the secretion of human bone marrow mesenchymal stem cells can effectively promote the proliferation and differentiation of rat bone marrow mesenchymal stem cells. Qi et al. (2016) combined human induced pluripotent stem cell-derived exosomes with β-tricalcium phosphate (β-TCP) scaffolds to repair rat skull defects. It was found that the repair effect of the exosome composite scaffolds was significantly better than that of the β-TCP scaffolds alone, and exosomes could promote the osteogenic differentiation of bone marrow mesenchymal stem cells by activating the PI3K/Akt signaling pathway. In this study, exosomes derived from M2 RAW264.7 cells induced upregulation of Runx2 and Collagen I expression in MC3T3-E1 cells, and the effect was significantly better than that in the macrophage co-culture group, indicating that exosomes play an important role in the process of osteoimmunity-promoting osseointegration. At the same time, we found that three miRNAs were upregulated and eight miRNAs were downregulated in M2 RAW264.7 cell-derived exosomes, and the corresponding target genes involved in the regulation of multiple signaling pathways, such as mTOR, AMPK and Wnt signaling pathways, which play an important role in the process of osseointegration. After RNA sequencing of exosomes derived from hMSCs induced by osteogenesis, Zhai et al. (2020) found that exosomes induce osteogenic differentiation through microRNA, among which the miRNAs HAS-Mir-146a-5p, HAS-Mir-503-5p, HAS-Mir-483-3P, and HAS-MIR) that contribute to bone formation. The upregulation of HAS-Mir-32-5p, HAS-Mir-133a-3P, and HAS-Mir-204-5p activated the PI3K/Akt and MAPK signaling pathways related to osteogenesis.

## Conclusion

In conclusion, we successfully isolated exosomes from RAW264.7 cells, which were induced to polarize to the M2 phenotype by preparing small-scale topography titanium disks. After co-culture with MC3T3-E1 cells, we found that exosomes significantly promoted the osteogenic differentiation and mineralization of MC3T3-E1 cells. Through RNA sequencing and gene analysis, we found differentially expressed microRNAs that targeted the signal pathways that may be related, such as mTOR, AMPK, Wnt, etc., and thus provide a reference for the mechanism of osteoimmunue regulation of implant osseointegration. The deficiency of this study is that the selection of RAW264.7 cells and MC3T3-E1 cells has certain limitations, and the related research is still insufficient. In the next research, we will focus on the exosome microRNA and its downstream key factors, and further study the molecular mechanism of osteoimmune effect on small-scale topography implant osseointegration through exosomes.

## Data Availability Statement

The datasets presented in this study can be found in online repositories. The names of the repository/repositories and accession number(s) can be found below: GSE175428, https://www.ncbi.nlm.nih.gov/geo/query/acc.cgi?acc=GSE175428.

## Author Contributions

HS, PY, and TZ conceived the project and designed experiments. TZ, MJ, and XY performed the experiments. TZ and MJ analyzed the data. TZ wrote the manuscript. All authors commented on the manuscript.

## Conflict of Interest

The authors declare that the research was conducted in the absence of any commercial or financial relationships that could be construed as a potential conflict of interest.
