# Supplementary figures and images for: Mechanism of Exosomes Involved in Osteoimmunity Promoting Osseointegration Around Titanium Implants With Small-Scale Topography
Source: Front Bioeng Biotechnol. 2021 Jul 15;9:682384. doi: 10.3389/fbioe.2021.682384 (PMC8320438; doi:10.3389/fbioe.2021.682384)

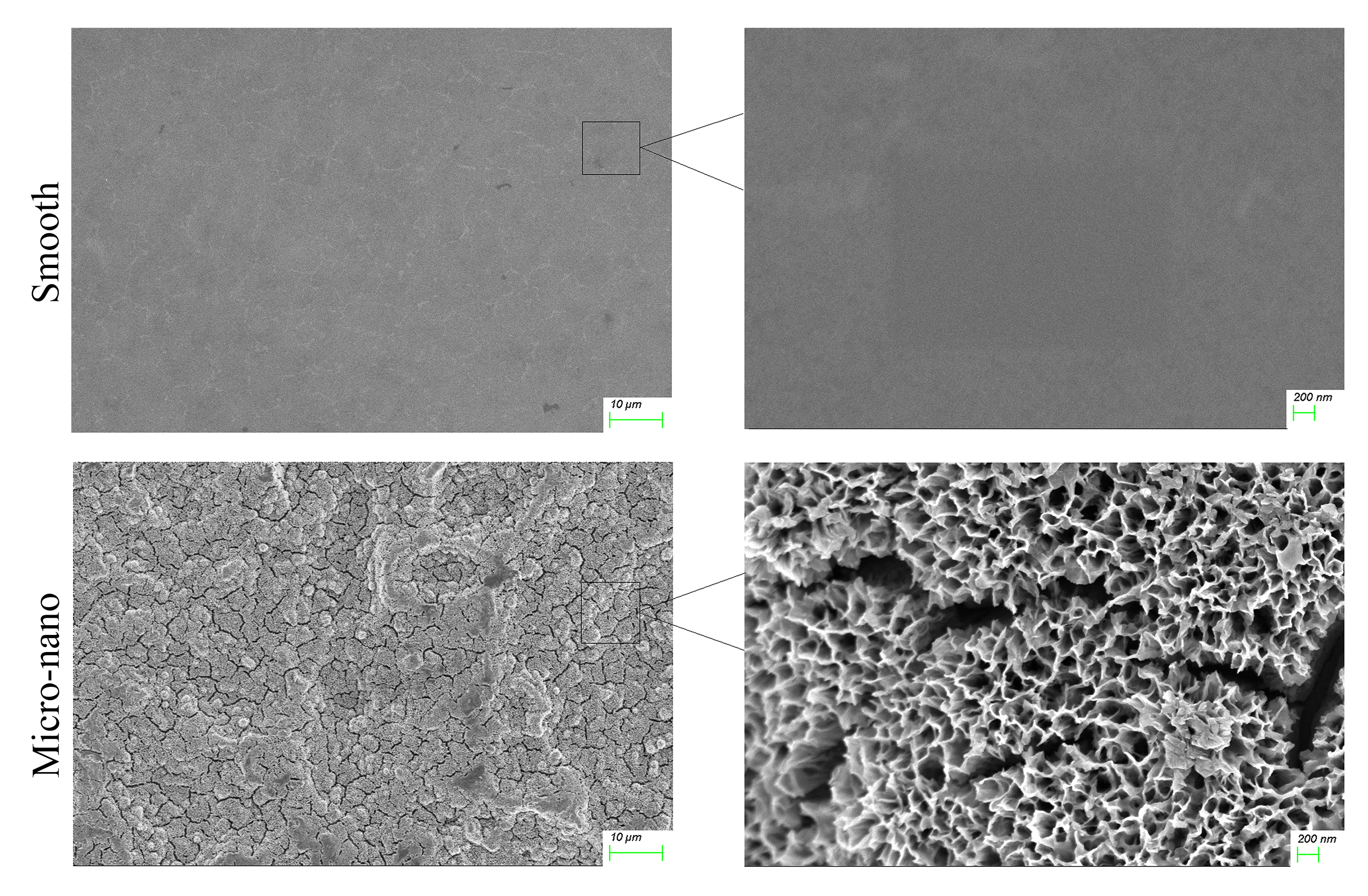

Supplement: Supplementary file 1 [file Image_1.TIF]

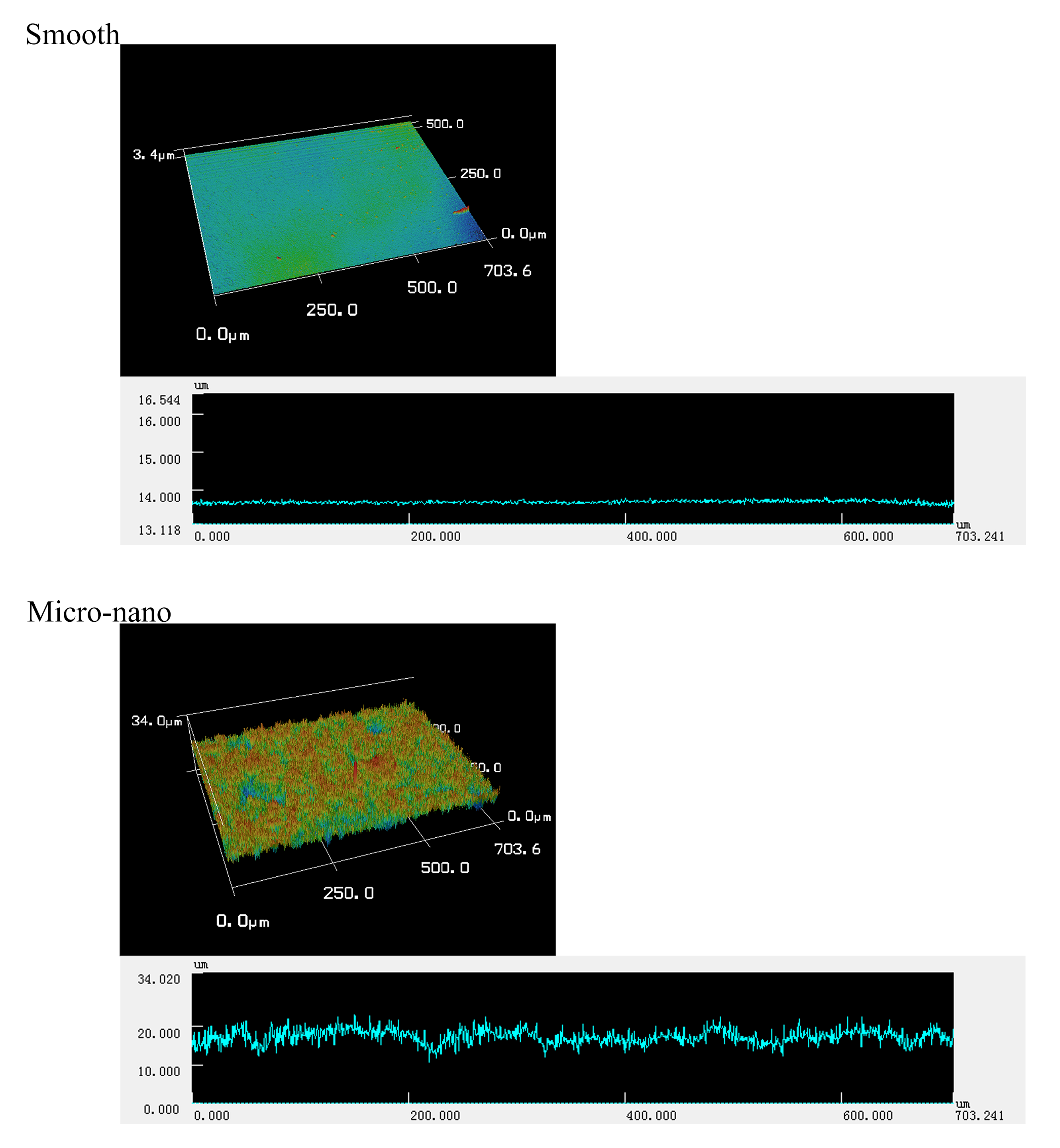

Supplement: Supplementary file 2 [file Image_2.TIF]
